# Pro-social 50-kHz ultrasonic communication in rats: post-weaning but not post-adolescent social isolation leads to social impairments—phenotypic rescue by re-socialization

**DOI:** 10.3389/fnbeh.2015.00102

**Published:** 2015-05-01

**Authors:** Dominik Seffer, Henrike Rippberger, Rainer K. W. Schwarting, Markus Wöhr

**Affiliations:** Behavioral Neuroscience, Experimental and Biological Psychology, Philipps-University of MarburgMarburg, Germany

**Keywords:** juvenile social isolation, affiliative behavior, rough-and-tumble play, schizophrenia, negative symptoms

## Abstract

Rats are highly social animals and social play during adolescence has an important role for social development, hence post-weaning social isolation is widely used to study the adverse effects of juvenile social deprivation and to induce behavioral phenotypes relevant to neuropsychiatric disorders, like schizophrenia. Communication is an important component of the rat's social behavior repertoire, with ultrasonic vocalizations (USV) serving as situation-dependent affective signals. High-frequency 50-kHz USV occur in appetitive situations and induce approach behavior, supporting the notion that they serve as social contact calls; however, post-weaning isolation effects on the behavioral changes displayed by the receiver in response to USV have yet to be studied. We therefore investigated the impact of post-weaning isolation on socio-affective information processing as assessed by means of our established 50-kHz USV radial maze playback paradigm. We showed that post-weaning social isolation specifically affected the behavioral response to playback of pro-social 50-kHz but not alarm 22-kHz USV. While group-housed rats showed the expected preference, i.e., approach, toward 50-kHz USV, the response was even stronger in short-term isolated rats (i.e., 1 day), possibly due to a higher level of social motivation. In contrast, no approach was observed in long-term isolated rats (i.e., 4 weeks). Importantly, deficits in approach were reversed by peer-mediated re-socialization and could not be observed after post-adolescent social isolation, indicating a critical period for social development during adolescence. Together, these results highlight the importance of social experience for affiliative behavior, suggesting a critical involvement of play behavior on socio-affective information processing in rats.

## Introduction

In social species, like humans and other mammals, early life experiences are crucial for the development of social and cognitive skills. Positive relationships between parent and child, as well as among peers promote appropriate acquisition of adult social competency. In contrast, disruptions of social attachment during critical developmental periods can lead to various behavioral alterations, including deficits in emotional regulation and social impairments, and are considered as non-specific risk factors for developing psychopathologies (Cicchetti and Toth, 1995; Veenema, 2009; Braun and Bock, 2011). In fact, child maltreatment, such as sexual/physical abuse and social neglect, contribute to various forms of impaired functioning characterized by social withdrawal, like the development of anxiety and depressive disorders, but also psychosis and schizophrenia (Brown et al., 1999; Lansford et al., 2002; Read et al., 2005; Gilbert et al., 2009; Spinhoven et al., 2010; Varese et al., 2012; DeRosse et al., 2014). This could possibly be due to adverse effects on social information processing, such as attention to social cues, consequently leading to inappropriate regulation of both negative affect and aggression (Dodge et al., 1990; Shackman and Pollak, 2014). For instance, children exposed to an extreme form of social deprivation while being raised in an orphanage have an increased risk for delayed cognitive and socio-affective development, along with mental health disorders. Specifically, they display attention deficits, hyperactivity, and increased anxiety, together with impaired emotional regulation and social attachment (Nelson et al., 2007; Bos et al., 2011). Moreover, their language development is delayed, with particular deficits in verbal skills (Frank et al., 1996).

In rats, maternal separation and post-weaning social isolation are widely used to study the adverse effects of early social deprivation. Importantly, rats are highly social animals and live in groups (Whishaw and Kolb, 2005) with a distinct hierarchy (Baenninger, 1966), offering optimal experimental conditions to investigate social behavior and communication. For instance, rats engage in social play (Panksepp, 1981) and cooperative behavior (Willner et al., 1989; Łopuch and Popik, 2011), display direct (Rutte and Taborsky, 2008) and generalized reciprocity (Rutte and Taborsky, 2007), and, as recently shown, prefer mutual rewards in a pro-social choice test, resulting in benefits not only for themselves but also for conspecifics (Hernandez-Lallement et al., 2015). Similarly, evidence for helping behavior and empathy has been provided (Ben-Ami Bartal et al., 2011; but see Silberberg et al., 2014). Play with peers emerges as one of the earliest forms of social behavior that is not directed to the mother. In rats, social play behavior (also called rough-and-tumble play or play fighting) mainly occurs after weaning and reaches its peak during the middle of the juvenile stage (Panksepp, 1981). Lack of play during the critical developmental period, due to post-weaning isolation, leads to a behavioral phenotype characterized by prominent social impairments in adulthood (Hol et al., 1999; van den Berg et al., 1999a). Most notably, long-term changes in behavior with relevance to various neuropsychiatric disorders can be observed, including hyperactivity in a novel environment, altered responses to drugs of abuse, impaired sensorimotor gating, cognitive inflexibility, increased aggressive behavior, and social withdrawal (Hall, 1998; Lapiz et al., 2003; Fone and Porkess, 2008).

For rats, communication is an important component in the social behavior repertoire. Rats produce and perceive calls in the ultrasonic range (so called ultrasonic vocalizations, USV). Based on their acoustic features, various USV types can be differentiated which serve distinct communicative functions, as situation-dependent affective signals (Portfors, 2007; Brudzynski, 2013; Wöhr and Schwarting, 2013). In juvenile and adult rats, a distinction is made between 22- and 50-kHz USV. Low-frequency 22-kHz USV occur in aversive situations, such as predator exposure, social defeat, and fear learning, and are thought to be reflecting a negative affective state of the sender. They serve as an alarm function and induce freezing behavior in the recipient. In contrast, high-frequency 50-kHz USV occur in appetitive situations, mostly social ones, such as rough-and-tumble play (Knutson et al., 1998), tickling (Panksepp and Burgdorf, 2000; Burgdorf and Panksepp, 2001), and sexual behavior (Sales, 1972), and are thought to be reflecting a positive affective state. In support for a communicative function, evidence was recently provided that juvenile 50-kHz USV promote and maintain playful social interactions (Himmler et al., 2014), while play behavior is reduced in pairs of devocalized rats (Kisko et al., 2015). Furthermore, it was repeatedly shown that playback of 50-kHz USV induces approach behavior in the recipient, supporting the notion that they serve as social contact calls (Wöhr and Schwarting, 2007, 2009, 2012; Willuhn et al., 2014).

Interestingly, there is evidence that post-weaning isolation affects USV production in rats. For instance, the rate of 50-kHz USV emission during social play and tickling is increased following isolation, possibly due to an increase in social motivation (Knutson et al., 1998; Panksepp and Burgdorf, 2000; Burgdorf and Panksepp, 2001). Furthermore, Inagaki et al. (2013) found a strong decrease in 50-kHz USV in male rats exposed to a sexually receptive female after prolonged isolation, likely reflecting their inability to recognize social cues and/or to respond appropriately. Finally, von Frijtag et al. (2002) observed that, in the presence of an aggressive resident, socially isolated male rats display impaired suppression of play behavior, suffer more harming attacks, and emit more 22-kHz USV than their group-housed counterparts. They suggested that 22-kHz USV production was dissociated from the sender's behavioral pattern and therefore lost its aggression-reducing communicative function.

In contrast, post-weaning isolation effects on the behavioral changes displayed by the receiver in response to USV have yet to be studied. Therefore, we investigated the impact of post-weaning isolation on socio-affective information processing as assessed by means of our established 50-kHz USV radial maze playback paradigm that allows to study pro-social ultrasonic communication in a reliable and highly standardized manner (Seffer et al., 2014). It was hypothesized that (I) post-weaning social isolation will impair approach behavior in response to playback of pro-social 50-kHz USV, that (II) this phenotype can be rescued by peer-mediated re-socialization, and that (III) isolation-induced deficits depend on the time period of isolation during development.

## Materials and methods

### Animals

Male Wistar rats (HsdCpb:WU, Harlan, Venray, The Netherlands) served as subjects and were housed in an animal room with a 12:12 h light/dark cycle (lights on 8–20 h) where the environmental temperature was maintained between 20 and 23°C (humidity: 30–50%). Lab chow (Altromin, Lage, Germany) and water (0.0004% HCl-solution) were available *ad libitum*. Rats were handled for three consecutive days prior to testing in a standardized way for 5 min. All experimental procedures were performed according to legal requirements of Germany and approved by the ethical committee of the local government (Regierungspräsidium Gießen, Germany).

### General overview

**Figure 1:**
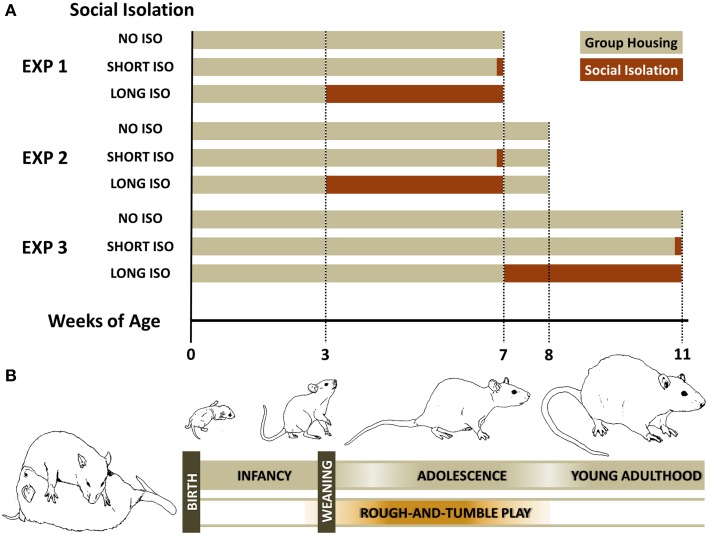
**Experimental design of the three studies to assess the impact of post-weaning social isolation on approach behavior induced by pro-social 50-kHz USV. (A)** Experimental designs: Experiment 1: Post-weaning Social Isolation—Effects; Experiment 2: Post-weaning Social Isolation—Rescue; and Experiment 3: Post-adolescent Social Isolation—Effects. **(B)** Schematic illustration of the rat developmental profile, highlighting the rough-and-tumble play period.

To assess the impact of post-weaning social isolation on approach behavior induced by pro-social 50-kHz USV, we used our 50-kHz USV radial maze playback paradigm (Seffer et al., 2014). In total, three experiments were conducted, all using a 3 × 3 design with the factors experimental housing condition and acoustic stimulus (Figure 1A). Nine experimental groups of *N* = 12 rats per experimental housing/acoustic stimulus combination were used. Rats were exposed to one of the following three acoustic stimuli: (I) pro-social 50-kHz USV; (II) alarm 22-kHz USV; or (III) background noise (NOISE). 22-kHz USV and NOISE were used to assure specificity of the effects of isolation on approach behavior induced by 50-kHz USV. Of note, Wistar rats were shown to have the ability to perceive acoustic stimuli in the audible and ultrasonic range (Borg, 1982).

### Experiment 1: post-weaning social isolation—effects

In Experiment 1, we exposed rats to one of the following three experimental housing conditions for 4 weeks: (I) group housing (NO ISO), with rats being housed in groups of six; (II) short-term social isolation (SHORT ISO), with rats housed in groups of six, but isolated 24 h prior testing; or (III) long-term social isolation (LONG ISO), with rats being isolated for 28 days prior testing. Rats were isolated as weanlings at about 3 weeks of age. At about 7 weeks of age (body weight: 246.44 ± 1.43 g; range: 208.5–276.5 g), they were exposed to (I) 50-kHz USV, (II) 22-kHz USV, or (III) NOISE.

### Experiment 2: post-weaning social isolation—rescue

In Experiment 2, we tested for phenotypic rescue by adding one additional week of peer rearing after being housed under one of the three experimental housing conditions for 4 weeks, i.e., (I) NO ISO, (II) SHORT ISO, or (III) LONG ISO, since recent findings demonstrated that social deficits displayed by a well-established autism model can be improved by peer intervention (Yang et al., 2011). As in Experiment 1, rats were isolated as weanlings at about 3 weeks of age. After one additional week of peer rearing, at about 8 weeks of age (body weight: 280.88 ± 2.01 g; range: 236.5–326.0 g), they were exposed to (I) 50-kHz USV, (II) 22-kHz USV, or (III) NOISE, as in Experiment 1.

### Experiment 3: post-adolescent social isolation—effects

In Experiment 3, we tested for the existence of a critical window for developing social deficits induced by post-weaning social isolation. To this aim, post-adolescent young adult rats at about 7 weeks of age, and after going through the rough-and-tumble play period (Figure 1B; Panksepp, 1981), were exposed to one of the three experimental housing conditions for 4 weeks, i.e., (I) NO ISO, (II) SHORT ISO, or (III) LONG ISO. At about 11 weeks of age (body weight: 383.93 ± 1.91 g; range: 349.0–444.0 g), they were exposed to (I) 50-kHz USV, (II) 22-kHz USV, or (III) NOISE, as in Experiment 1.

### Experimental housing

Group housing was conducted in polycarbonate Macrolon type IV cages (380 × 200 × 590 mm, plus high stainless steel covers) and isolation rearing in Macrolon type III cages (265 × 150 × 425 mm, plus high stainless steel covers), both filled with Tapvei peeled aspen bedding (indulab ag, Gams, Switzerland).

### Experimental setting

**Figure 2:**
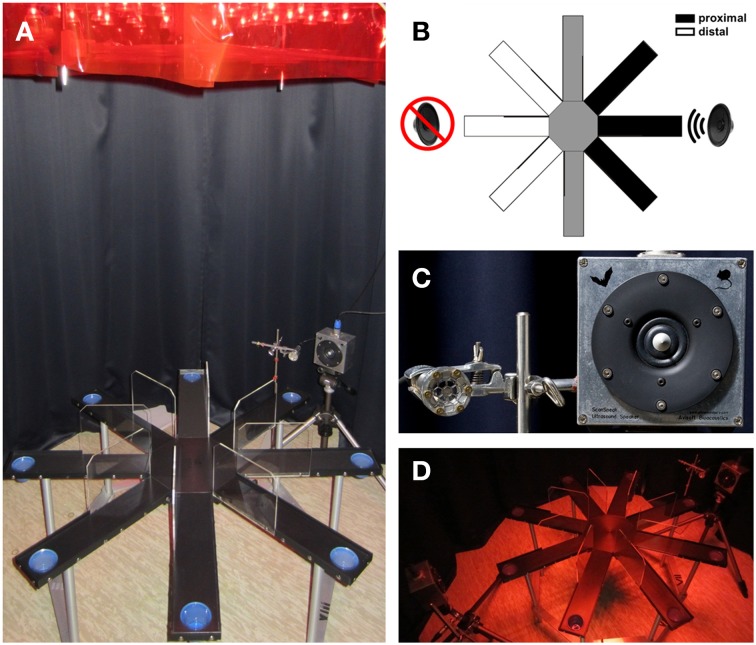
**Setup of the 50-kHz USV radial maze playback paradigm. (A)** Elevated eight arm radial maze equipped with two ultrasonic microphones and two ultrasonic loudspeakers positioned opposite to each other (only one ultrasonic microphone and the active loudspeaker are shown). The rat's behavioral responses during playback of acoustic stimuli were recorded with a video camera positioned above the radial maze. **(B)** Schematic illustration of the radial maze, depicting area definitions to assess approach behavior, with arms close to the loudspeaker denoted as proximal, central arms as neutral, and opposite arms as distal. **(C)** Acoustic stimuli were presented by an ultrasonic loudspeaker (sampling rate: 192 kHz; 16 bit), with accurate playback of acoustic stimuli being confirmed by means of ultrasonic microphones. **(D)** Testing was performed under dim red light.

Playback of acoustic stimuli was conducted under dim red light (~10 lux) on a radial eight arm maze made of black plastic, elevated 52 cm above the floor (Figures 2A–D). The arms (9.8 × 40.5 cm) extend radially from a central platform (diameter: 24 cm). Acoustic stimuli were presented through an ultrasonic loudspeaker (ScanSpeak, Avisoft Bioacoustics, Berlin, Germany) using an external sound card with a sampling rate of 192 kHz (Fire Wire Audio Capture FA-101, Edirol, London, UK). The loudspeaker had a frequency range of 1–120 kHz with a relatively flat frequency response (±12 dB) between 15 and 80 kHz and it was placed 20 cm away from the end of one arm. An additional, but inactive ultrasonic loudspeaker was arranged symmetrically at the opposite arm as visual control. Rats were tested in a testing room with no experimenter or other rats present. Stimulus application and animal observation was performed in a separate control room. Behavioral tests were conducted between 8 and 18 h. Before each test, behavioral equipment was cleaned (0.1% acetic acid solution) and dried.

### Acoustic stimuli

**Figure 3:**
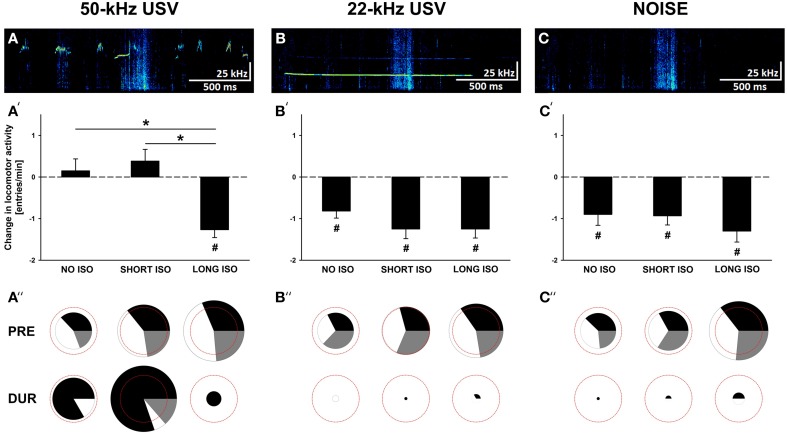
**Post-weaning social isolation induces behavioral inhibition in response to pro-social 50-kHz USV similar to alarm 22-kHz USV and NOISE. (A–C)** Exemplary spectrograms of acoustic stimuli used for playback, namely **(A)** 50-kHz USV, **(B)** 22-kHz USV, and **(C)** background noise (NOISE). **(A′–C′)** Bar graphs depicting changes in locomotor activity as assessed by means of total arm entries per min [entries/min] displayed by rats exposed to 4 weeks of NO ISO (left), SHORT ISO (middle), and LONG ISO (right), post-weaning, in response to **(A′)** 50-kHz USV, **(B′)** 22-kHz USV, and **(C′)** NOISE, in Experiment 1: Post-weaning Social Isolation—Effects. **(A″–C″)** Pie charts depicting changes in stimulus-directed locomotor activity as assessed by means of numbers of proximal (black), neutral (gray), and distal (white) arm entries displayed as percentages by rats exposed to 4 weeks of NO ISO (left), SHORT ISO (middle), and LONG ISO (right), post-weaning, during the baseline period (5 min; upper row; PRE) and during playback (1 min; lower row; DUR) in response to **(A″)** 50-kHz USV, **(B″)** 22-kHz USV, and **(C″)** NOISE, in Experiment 1: Post-weaning Social Isolation—Effects. The red dashed circles represent 100% of the total number of arm entries during the baseline period (average over all nine experimental groups). The sizes of the circles depicting proximal (black), neutral (gray), and distal (white) arm entries represent total number of arm entries as percentage of 100%. ^*^p < 0.050 compared to LONG ISO; ^#^*p* < 0.050 compared to baseline locomotor activity.

Rats were exposed to 5 min of playback of one of the following three acoustic stimuli: (I) pro-social 50-kHz USV, (II) alarm 22-kHz USV, and (III) background noise (NOISE; for details see (Wöhr and Schwarting, 2007, 2009, 2012; Seffer et al., 2014; Willuhn et al., 2014; Figures 3A–C, Supplementary Figures 2A–C, 3A–C). Briefly, playback of 50-kHz USV consisted of 1105 calls, including flat and frequency-modulated calls, recorded from an adult male Wistar rat during exploration of a cage containing scents from a recently separated cage mate (for setting and recording see Wöhr et al., 2008). This recording context had been chosen since playback of 50-kHz USV recorded during tickling or exploration of an empty cage had no or only very moderate effects on the behavior displayed by the receiver (Burman et al., 2007; Endres et al., 2007). Average acoustic call parameters were (mean ± SEM): call duration: 0.07 ± 0.01 s; peak frequency: 61.24 ± 1.75 kHz; frequency modulation: 31.68 ± 4.62 kHz. Besides 50-kHz USV, playback contained background noise, produced by the rat while exploring the cage. Playback of 22-kHz USV consisted of 145 calls recorded from a male Wistar rat which had received electric foot-shocks before, but not during recording (for setting and recording see Wöhr et al., 2005). Average acoustic call parameters were: call duration: 1.18 ± 0.06 s; peak frequency: 23.61 ± 0.07 kHz; frequency modulation: 1.90 ± 0.09 kHz. To control for background noise as present in the 50-kHz USV stimulus, background noise was added to the 22-kHz USV stimulus. Background noise alone (NOISE), i.e., without USV, served to control for unspecific effects, i.e., novelty-induced changes in behavior not linked to the communicative function of 50-kHz USV. All stimuli were presented with a sampling rate of 192 kHz in 16 bit format at ~69 dB, with the exception of NOISE that was presented at ~50 dB (measured from a distance of 40 cm).

### Experimental procedure

Rats were placed onto the central platform of the radial maze with their body axis at an angle of 90° to the two ultrasonic loudspeakers. After an initial habituation phase of 15 min where no acoustic stimuli were presented, the rat was exposed to one of the three acoustic stimuli for 5 min.

### Behavioral recording and analysis

Behavior was monitored by a video camera (Panasonic WV-BP 330/GE, Hamburg, Germany) from about 150 cm above the radial maze, which fed into an external multimedia hard drive (ScreenPlay Pro HD, Iomega, San Diego, CA, USA). For behavioral analysis, a trained observer scored the videos for the following parameters (Seffer et al., 2014): (I) total number of arm entries, (II) number of entries into the three arms proximal to or distal from the active ultrasonic loudspeaker, and (III) the time spent on proximal and distal arms. An arm entry was counted when all four paws were in the arm. Total number of arm entries served as measure for general locomotor activity. Proximal vs. distal arm entries and the time spent thereon served as measures for stimulus-directed locomotor activity. Behavioral analysis was focused on the first min of acoustic stimulus exposure, since behavioral changes in response to 50-kHz USV are short-lasting, with most prominent changes occurring during the first min of playback (Wöhr and Schwarting, 2012; Willuhn et al., 2014; Supplementary Figures 1A,B).

### Usv recording and analysis

Accurate playback of acoustic stimuli was confirmed by means of two UltraSoundGate Condenser Microphones (CM16; Avisoft Bioacoustics) placed opposite to each other, 20 cm away from the radial maze (Seffer et al., 2014). For acoustical analysis, recordings (sampling rate: 250 kHz; format: 16 bit) were transferred to SASLab Pro (version 4.38; Avisoft Bioacoustics) and a fast Fourier transform was conducted (512 FFT-length, 100% frame, Hamming window and 75% time window overlap; Seffer et al., 2014). Importantly, no substantial USV production (<1/min on average) by the rats under study was detected (data not shown).

### Statistical analysis

General locomotor activity during the baseline period (PRE: total arm entries 5 min prior playback; average per min) was analyzed using ANOVAs with the between-subject factor experimental housing condition (NO ISO, SHORT ISO, vs. LONG ISO). To assess the effects of experimental housing conditions on stimulus-induced locomotor activity, ANOVAs for repeated measurements were calculated with the within-subject factor stimulus presentation before and during playback; average per min) and the between-subject factor experimental housing condition (NO ISO, SHORT ISO, vs. LONG ISO) for 50-kHz USV, 22-kHz USV, and NOISE. To directly compare the latter, ANOVAs with the between-subject factor stimulus type were used. ANOVAs were followed by LSD *post-hoc* or paired *t*-tests when appropriate (*p* < 0.050). Paired *t*-tests were also used to compare proximal and distal arm entries and the time spent thereon before and during playback. Three rats had to be excluded from data analysis due to technical problems (Experiment 1: *N* = 1 from the NO ISO/22-kHz USV combination; Experiment 2: *N* = 1 from the SHORT ISO/50-kHz USV combination; Experiment 3: *N* = 1 from the NO ISO/NOISE combination). Data are presented as mean ± SEM. A *p*-value of < 0.050 was considered statistically significant.

## Results

### Experiment 1: post-weaning social isolation—effects

To assess the impact of post-weaning isolation on approach behavior induced by 50-kHz USV, we used our 50-kHz USV radial maze playback paradigm (Seffer et al., 2014) and assessed approach in post-weaning rats exposed to (I) NO ISO, (II) SHORT ISO, or (III) LONG ISO.

In response to all three acoustic stimuli presented, i.e., pro-social 50-kHz USV, alarm 22-kHz USV, and NOISE, changes in locomotor activity, as assessed by means of total arm entries, were detected (Figures 3A′–C′). While 22-kHz USV and NOISE led to behavioral inhibition irrespective of experimental housing condition [main effect stimulus presentation: *F*_(1, 32)_ = 82.719; *p* < 0.001; interaction housing × stimulus presentation: NS; and main effect stimulus presentation: *F*_(1, 32)_ = 52.538; *p* < 0.001; interaction housing × stimulus presentation: NS, respectively], the behavioral response to 50-kHz USV was strongly affected by post-weaning isolation [main effect stimulus presentation: NS; interaction housing × stimulus presentation: *F*_(1, 32)_ = 12.147; *p* < 0.001]. Specifically, rats exposed to LONG ISO displayed behavioral inhibition in response to 50-kHz USV [*t*_(11)_ = 6.680; *p* < 0.001], whereas NO ISO or SHORT ISO maintained their level of locomotor activity [*t*_(11)_ = 0.526; *p* = 0.609 and *t*_(11)_ = 1.358; *p* = 0.202, respectively], with both being significantly different from LONG ISO (*p* < 0.001 and *p* < 0.001, respectively). Importantly, LONG ISO rats displayed a level of behavioral inhibition in response to 50-kHz USV that was similar to the one seen in rats exposed to 22-kHz USV or NOISE (main effect stimulus type: NS).

Behavioral inhibition in response to 22-kHz USV and NOISE was typically reflected by a reduction in both, proximal and distal arm entries (not shown in detail; Figures 3B″,C″). In contrast, locomotor activity displayed by NO ISO and SHORT ISO rats in response to 50-kHz USV was clearly directed toward the sound source, as reflected by an increase in the number of proximal arm entries [*t*_(11)_ = 2.820; *p* = 0.017 and *t*_(11)_ = 3.336; *p* = 0.007, respectively], often paralleled by a decrease in distal arm entries [*t*_(11)_ = 0.527; *p* = 0.609 and *t*_(11)_ = 3.960; *p* = 0.002, respectively], whereas in LONG ISO rats proximal arm entries did not increase, with distal arm entries being reduced due to behavioral inhibition [*t*_(11)_ = 0.924; *p* = 0.375 and *t*_(11)_ = 9.544; *p* < 0.001, respectively; Figure 3A″]. In general, locomotor activity during the baseline period was increased in rats exposed to LONG ISO [main effect housing: *F*_(2, 104)_ = 6.816; *p* = 0.002; LONG ISO vs. NO ISO: *p* < 0.001; LONG ISO vs. SHORT ISO: *p* = 0.059; SHORT ISO vs. NO ISO: *p* = 0.076]. This effect was also detectable in the subset of rats exposed to the 50-kHz USV stimulus condition, as reflected in the size of the pie charts [main effect housing: *F*_(2, 33)_ = 3.702; *p* = 0.035; LONG ISO vs. NO ISO: *p* = 0.011; LONG ISO vs. SHORT ISO: *p* = 0.355; SHORT ISO vs. NO ISO: *p* = 0.091].

**Figure 4:**
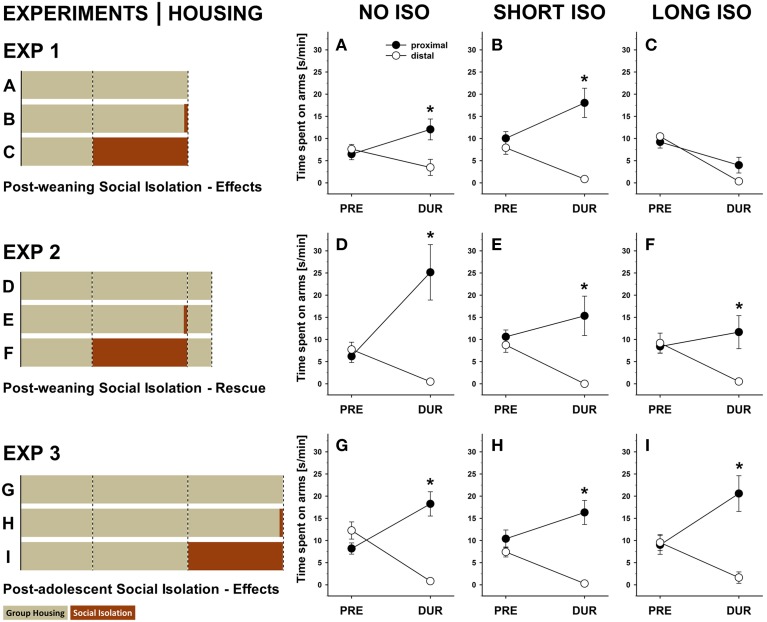
**Post-weaning but not post-adolescent social isolation leads to a lack of approach behavior in response to pro-social 50-kHz USV that can be reversed by re-socialization: within-group comparisons. (A–C)** Line graphs depicting changes in side preferences as assessed by the time spent [s/min] on proximal (black) and distal (white) arms displayed by rats exposed to 4 weeks of **(A)** NO ISO, **(B)** SHORT ISO, and **(C)** LONG ISO, post-weaning, during the baseline period (5 min; PRE) and during playback (1 min; DUR) in response to 50-kHz USV in Experiment 1: Post-weaning Social Isolation—Effects. **(D–F)** Line graphs depicting changes in side preferences as assessed by the time spent [s/min] on proximal (black) and distal (white) arms displayed by rats exposed to 4 weeks of **(D)** NO ISO, **(E)** SHORT ISO, and **(F)** LONG ISO, post-weaning, plus one additional week of peer rearing, during the baseline period (5 min; PRE) and during playback (1 min; DUR) in response to 50-kHz USV in Experiment 2: Post-weaning Social Isolation—Rescue. **(G–I)** Line graphs depicting changes in side preferences as assessed by the time spent [s/min] on proximal (black) and distal (white) arms displayed by rats exposed to 4 weeks of **(G)** NO ISO, **(H)** SHORT ISO, and **(I)** LONG ISO, post-adolescent, during the baseline period (5 min; PRE) and during playback (1 min; DUR) in response to 50-kHz USV in Experiment 3: Post-adolescent Social Isolation—Effects. ^*^p < 0.050 compared to distal arm time.

Approach behavior induced by 50-kHz USV was not only reflected by proximal and distal arm entries but also by the time spent thereon. In NO ISO rats, the proximal time increased [*t*_(11)_ = 2.920; *p* = 0.014], while the distal time decreased [*t*_(11)_ = 2.450; *p* = 0.032], resulting in a significant preference toward the stimulus source during 50-kHz USV playback [*t*_(11)_ = 3.621; *p* = 0.004; Figure 4A]. Likewise, in SHORT ISO rats the proximal time increased [*t*_(11)_ = 2.526; *p* = 0.028], while the distal time decreased [*t*_(11)_ = 5.947; *p* < 0.001], again resulting in a significant preference toward the stimulus source during 50-kHz USV playback [*t*_(11)_ = 5.641; *p* < 0.001; Figure 4B]. However, this response pattern was not seen in LONG ISO rats when exposed to 50-kHz USV, since they displayed avoidance behavior and spent less time on both, proximal and distal arms [*t*_(11)_ = 2.889; *p* = 0.015 and *t*_(11)_ = 14.581; *p* < 0.001, respectively], and no significant preference toward the stimulus source during 50-kHz USV playback [*t*_(11)_ = 2.040; *p* = 0.066; Figure 4C]. No significant preferences were seen before playback (all *p*-values > 0.050).

Together, Experiment 1 shows that post-weaning isolation has a strong impact on approach behavior elicited by 50-kHz USV. Consistent with previous studies (Wöhr and Schwarting, 2007, 2009, 2012; Willuhn et al., 2014), approach is seen in NO ISO and SHORT ISO rats. Rats exposed to LONG ISO, however, respond to 50-kHz USV by behavioral inhibition, similar to the one seen in response to 22-kHz USV or NOISE, resulting in a complete lack of approach behavior. Importantly, post-weaning isolation specifically affected the behavioral response to 50-kHz USV and did not change the responses seen in rats exposed to 22-kHz USV or NOISE.

### Experiment 2: post-weaning social isolation—rescue

To test whether it is possible to revert the deficits in approach behavior induced by post-weaning isolation, we assessed approach in post-weaning rats exposed to (I) NO ISO, (II) SHORT ISO, or (III) LONG ISO, followed by an additional week of peer rearing.

As in Experiment 1, changes in locomotor activity were detected in response to all three acoustic stimuli presented (Supplementary Figures 2A′–C′). Alarm 22-kHz USV led to behavioral inhibition, irrespective of experimental housing condition [main effect stimulus presentation: *F*_(1, 33)_ = 35.597; *p* < 0.001; interaction housing × stimulus presentation: NS]. Behavioral inhibition was also seen in response to NOISE in all experimental housing conditions, yet most prominently in LONG ISO rats [main effect stimulus presentation: *F*_(1, 32)_ = 100.938; *p* < 0.001; interaction housing × stimulus presentation: *F*_(1, 32)_ = 5.704; *p* = 0.008; as compared to NO ISO: *p* = 0.003; and SHORT ISO: *p* = 0.014]. Also, the behavioral response to pro-social 50-kHz USV was affected by post-weaning isolation [main effect stimulus presentation: NS; interaction housing × stimulus presentation: *F*_(1, 32)_ = 4.968; *p* = 0.013]. Specifically, rats exposed to NO ISO slightly increased their locomotor activity in response to 50-kHz USV [*t*_(11)_ = 1.987; *p* = 0.072], whereas, after one additional week of group housing, SHORT ISO but not LONG ISO rats slightly decreased their locomotor activity [*t*_(10)_ = 2.191; *p* = 0.053 and *t*_(11)_ = 0.685; *p* = 0.508, respectively], with LONG ISO not being significantly different from both, NO ISO and SHORT ISO, anymore (*p* = 0.072 and *p* = 0.199, respectively). Importantly, after the additional week of group housing, prior LONG ISO rats did not display a level of behavioral inhibition in response to 50-kHz USV that is similar to the one seen in rats exposed to 22-kHz USV or NOISE [main effect stimulus type: *F*_(1, 34)_ = 12.710; *p* < 0.001; *p* = 0.055 and *p* < 0.001, respectively]. Such a behavioral inhibition was shown by LONG ISO rats in Experiment 1 not exposed to an additional week of group housing.

As in Experiment 1, behavioral inhibition in response to 22-kHz USV and NOISE was typically reflected by a reduction in both, proximal and distal arm entries (not shown in detail; Supplementary Figures 2B″,C″). In contrast, locomotor activity displayed by NO ISO and SHORT ISO rats, after one additional week of group housing, in response to 50-kHz USV was clearly directed toward the sound source, as typically reflected by an increase in the number of proximal arm entries [*t*_(11)_ = 2.721; *p* = 0.020 and *t*_(10)_ = 0.593; *p* = 0.566, respectively], paralleled by a decrease in distal arm entries [*t*_(11)_ = 4.750; *p* = 0.001 and *t*_(10)_ = 5.043; *p* = 0.001, respectively]. Most importantly, LONG ISO rats, after one additional week of group housing, also displayed a slight increase in proximal arm entries [*t*_(11)_ = 1.984; *p* = 0.073], paralleled by a decrease in distal arm entries [*t*_(11)_ = 4.899; *p* < 0.001; Supplementary Figure 2A″].

Furthermore, in NO ISO rats the proximal time increased [*t*_(11)_ = 3.088; *p* = 0.010], while the distal time decreased [*t*_(11)_ = 4.894; *p* < 0.001], resulting in a significant preference toward the stimulus source during 50-kHz USV playback [*t*_(11)_ = 3.874; *p* = 0.003; Figure 4D]. Likewise, SHORT ISO rats, after one additional week of group housing, displayed a preference toward the stimulus source during 50-kHz USV playback [preference: *t*_(10)_ = 3.448; *p* = 0.006; proximal time: *t*_(10)_ = 1.063; *p* = 0.313; distal time: *t*_(11)_ = 5.287; *p* < 0.001; Figure 4E). Most importantly, however, LONG ISO rats, after one additional week of group housing, also displayed a preference toward 50-kHz USV playback [preference: *t*_(11)_ = 5.957; *p* = 0.009; proximal time: *t*_(10)_ = 0.879; *p* = 0.398; distal time: *t*_(11)_ = 4.014; *p* = 0.002; Figure 4F]. No significant preferences were seen before playback (all *p*-values >0.050).

Together, Experiment 2 shows that deficits in approach behavior induced by post-weaning isolation can be rescued by additional exposure to 1 week of peer rearing. This is in line with recent findings demonstrating that social deficits can be improved by peer intervention in a well-established autism model (Yang et al., 2011).

### Experiment 3: post-adolescent social isolation—effects

To test whether the deficits in approach behavior induced by post-weaning social isolation occur specifically during the juvenile developmental period, we repeated the experiment and assessed approach in post-adolescent rats exposed to (I) NO ISO, (II) SHORT ISO, or (III) LONG ISO.

As before, changes in locomotor activity were detected in response to alarm 22-kHz USV and NOISE, yet, in Experiment 3, no effect of pro-social 50-kHz USV was detected (Supplementary Figures 3A′–C′). Again, 22-kHz USV and NOISE led to behavioral inhibition irrespective of experimental housing condition [main effect stimulus presentation: *F*_(1, 33)_ = 53.598; *p* < 0.001; interaction housing × stimulus presentation: NS and main effect stimulus presentation: *F*_(1, 32)_ = 30.517; *p* < 0.001; interaction housing × stimulus presentation: NS, respectively], while no change in locomotor activity in response to 50-kHz USV was detected (main effect stimulus presentation: NS; interaction housing × stimulus presentation: NS). Importantly, post-adolescent LONGISO rats did not display a level of behavioral inhibition in response to 50-kHz USV that is similar to the one seen in rats exposed to 22-kHz USV or NOISE [main effect stimulus type: *F*_(1, 35)_ = 4.663; *p* = 0.016; *p* = 0.029 and *p* = 0.007, respectively]. Such a behavioral inhibition was shown by LONG ISO rats in Experiment 1 after post-weaning isolation.

Again, as in Experiments 1 and 2, behavioral inhibition in response to 22-kHz USV and NOISE was typically reflected by a reduction in both, proximal and distal arm entries (not shown in detail; Supplementary Figures 3B′,C′). In contrast, locomotor activity in response to 50-kHz USV was clearly directed toward the sound source, as reflected by an increase in the number of proximal arm entries, in post-adolescent rats of all three experimental housing conditions, namely NO ISO [*t*_(11)_ = 2.871; *p* = 0.015], SHORT ISO [*t*_(11)_ = 3.490; *p* = 0.005], and LONG ISO [*t*_(11)_ = 3.874; *p* = 0.003], typically paralleled by a decrease in distal arm entries [*t*_(11)_ = 3.442; *p* = 0.006, *t*_(11)_ = 3.149; *p* = 0.009, and *t*_(11)_ = 1.590; *p* = 0.140, respectively; Supplementary Figure 3A′].

Furthermore, in post-adolescent NO ISO rats the proximal time increased [*t*_(11)_ = 3.336; *p* = 0.007], while the distal time decreased [*t*_(11)_ = 6.416; *p* < 0.001], resulting in a significant preference toward the stimulus source during 50-kHz USV playback [*t*_(11)_ = 5.957; *p* < 0.001; Figure 4G]. Likewise, post-adolescent SHORT ISO rats displayed a preference toward the stimulus source during 50-kHz USV playback [preference: *t*_(11)_ = 5.566; *p* < 0.001; proximal time: *t*_(11)_ = 1.063; *p* = 0.313; distal time: *t*_(11)_ = 5.287; *p* < 0.001; Figure 4H]. Most importantly, however, post-adolescent LONG ISO rats also displayed a preference toward 50-kHz USV playback [preference: *t*_(11)_ = 4.726; *p* = 0.001; proximal time: *t*_(11)_ = 3.709; *p* = 0.003; distal time: *t*_(11)_ = 3.903; *p* = 0.002; Figure 4I]. No significant preferences were seen before playback (all *p*-values >0.050).

Together, Experiment 3 shows that post-adolescent isolation did not affect approach behavior elicited by 50-kHz USV. This indicates that the deficits in approach induced by post-weaning isolation is specific for the juvenile period, while no such effects are seen in post-adolescents, i.e., young adults after their rough-and-tumble play period (Panksepp, 1981).

### Comparison across experiments

**Figure 5:**
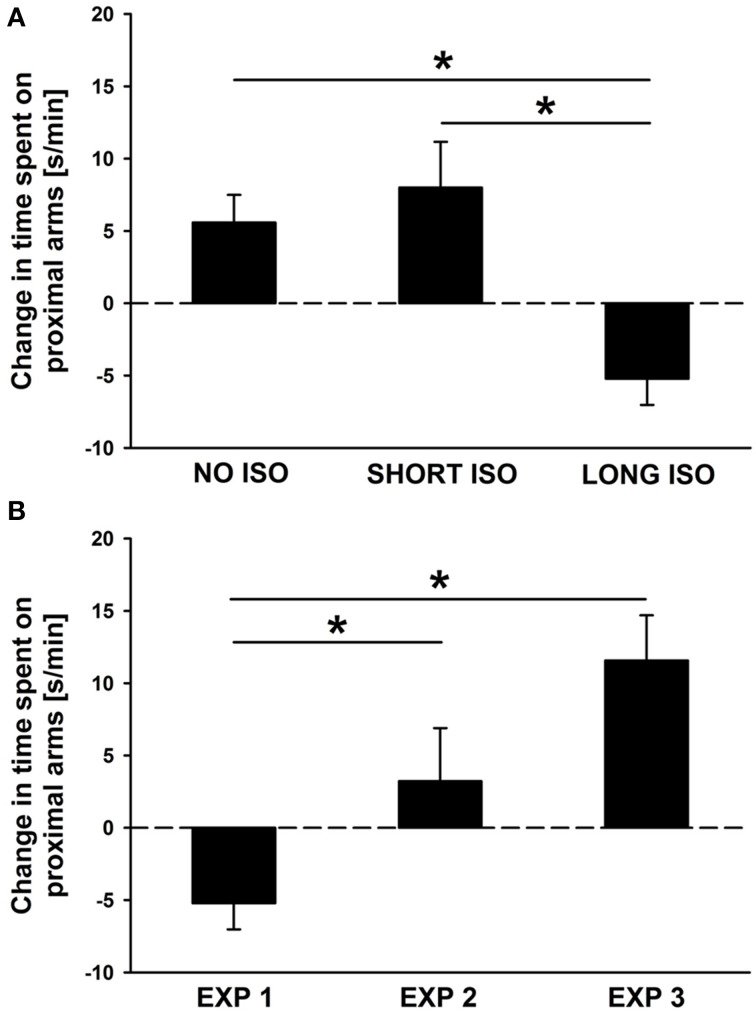
**Post-weaning but not post-adolescent social isolation leads to a lack of approach behavior in response to pro-social 50-kHz USV that can be reversed by re-socialization: between-group comparisons. (A)** Bar graphs depicting changes in side preferences as assessed by the time spent [s/min] on proximal arms during playback in response to 50-kHz USV (1 min) vs. during the baseline period (5 min), displayed by rats exposed to 4 weeks of NO ISO (left), SHORT ISO (middle), and LONG ISO (right), post-weaning, in Experiment 1: Post-weaning Social Isolation—Effects. ^*^p < 0.050 compared to LONG ISO. **(B)** Bar graphs depicting changes in side preferences as assessed by the time spent [s/min] on proximal arms during playback in response to 50-kHz USV (1 min) vs. during the baseline period (5 min), displayed by rats exposed to 4 weeks of LONG ISO, post-weaning, in Experiment 1: Post-weaning Social Isolation—Effects (left), 4 weeks of LONG ISO, post-weaning, plus one additional week of peer rearing in Experiment 2: Post-weaning Social Isolation—Rescue (middle), 4 weeks of LONG ISO, post-adolescent, in Experiment 3: Post-adolescent Social Isolation—Effects (right). ^*^p < 0.050 compared to EXP 1.

In Experiment 1 (effects of post-weaning social isolation), NO ISO and SHORT ISO rats displayed approach behavior in response to playback of pro-social 50-kHz USV, as reflected in an increase in the time spent on proximal arms, whereas LONG ISO rats displayed social avoidance, as reflected in a decrease in proximal arm times, resulting in a significant difference between experimental housing conditions [main effect housing: *F*_(1, 35)_ = 8.770; *p* = 0.001; Figure 5A]. Specifically, LONG ISO rats differed from NO ISO and SHORT ISO rats (*p* = 0.003 and *p* < 0.001, respectively), with the latter ones not differing from each other (*p* = 0.478). Importantly, when comparing LONG ISO rats between the three experiments, LONG ISO rats exposed to post-weaning isolation displaying social avoidance differed from the LONG ISO rats of the other two experiments [main effect housing: *F*_(1, 35)_ = 7.997; *p* = 0.001; Figure 5B], namely LONG ISO rats exposed to post-weaning isolation after one additional week of group housing (*p* = 0.028; one-tailed) and LONG ISO rats exposed to post-adolescent social isolation (*p* < 0.001; one-tailed), both displaying approach behavior. However, approach tended to be stronger in LONG ISO rats exposed to post-adolescent isolation than in LONG ISO rats exposed to post-weaning isolation after one additional week of group housing (*p* = 0.055). This indicates that the rescue by social peer intervention was only partial. Of note, the trend for stronger approach behavior in LONG ISO rats exposed to post-adolescent isolation is unlikely to be explained by age-differences, as approach behavior diminishes with age (Wöhr and Schwarting, 2007).

## Discussion

Post-weaning social isolation had a strong impact on approach behavior elicited by pro-social 50-kHz USV, as shown in Experiment 1. Approach was seen in group-housed and short-term isolated rats, which is consistent with previous studies (Wöhr and Schwarting, 2007, 2009, 2012; Willuhn et al., 2014). Rats exposed to long-term isolation, however, responded to 50-kHz USV by behavioral inhibition, similar to that seen in rats exposed to alarm 22-kHz USV or NOISE, resulting in a complete lack of approach behavior. General locomotor activity during the baseline period was increased in long-term isolated rats, in line with other studies reporting hyperactivity in response to a novel environment after post-weaning isolation (Hall, 1998; Lapiz et al., 2003; Fone and Porkess, 2008; Gaskin et al., 2014). Therefore, deficits in approach behavior cannot be due to a general lack of locomotor activity. Most notably, as shown in Experiments 2 and 3, respectively, such impairments could be rescued by exposure to one additional week of peer rearing and were not seen after post-adolescent social isolation.

Importantly, post-weaning isolation specifically affected the behavioral response to 50-kHz USV and did not change the responses to 22-kHz USV or NOISE. Playback of 22-kHz USV consistently led to behavioral inhibition in all three experiments, irrespective of experimental housing condition, i.e., in nine independent groups of rats. This is in line with previous studies, supporting the notion that they serve an alarm function (Burman et al., 2007; Endres et al., 2007; Wöhr and Schwarting, 2007, 2010). However, what appears surprising is that NOISE also led to behavioral inhibition, challenging the view that 22-kHz USV specifically induce behavioral inhibition as background noise was also present during 22-kHz USV playback. Future studies specifically focusing on behavioral inhibition induced by 22-kHz USV, thus appear requisite. In particular, a detailed analysis of freezing behavior might allow a more sensitive assessment of minor differences in the level of behavioral inhibition induced by 22-kHz USV and NOISE, as compared to the measure applied here, namely total number of arm entries. In the present context, however, it needs to be emphasized that 50-kHz USV playback consistently led to approach behavior, despite the presence of background noise, with only one exception, namely rats exposed to post-weaning long-term isolation. In future studies, it would be interesting to assess whether deficits in approach behavior following post-weaning social isolation are also seen in response to related acoustic stimuli, such as artificially/computer-generated 50-kHz sine wave tones.

Our present finding that post-weaning long-term isolation results in a complete lack of approach behavior in the receiver when exposed to 50-kHz USV, is in line with a number of studies reporting isolation-induced social deficits in rats, like reduced social activity, including approach, anogenital sniffing, and reciprocal interaction (Meaney and Stewart, 1979; Hol et al., 1999; van den Berg et al., 1999a,b,c; Kokare et al., 2010; Möller et al., 2011, 2013; but see Ferdman et al., 2007; Han et al., 2011), as well as heightened aggression (Wongwitdecha and Marsden, 1996; Vale and Montgomery, 1997; Meng et al., 2010; Wall et al., 2012; Grotewold et al., 2014), which is possibly due to impaired communication (Tóth et al., 2008). In fact, besides social behavior, post-weaning long-term isolation was also shown to affect communication. Specifically, male rats were found to emit fewer 50-kHz USV when exposed to a sexually receptive female, probably reflecting their inability to recognize social cues and/or to respond appropriately (Inagaki et al., 2013). Furthermore, it was reported that isolation-rearing leads to a dissociation of 22-kHz USV production from the sender's behavioral pattern, possibly resulting in a loss of its aggression-reducing communicative function (von Frijtag et al., 2002). In contrast, the receiver's response to 22-kHz USV was not affected by social isolation in our experiments, suggesting that they were either innately recognized as alarm calls or had gained alarm signal value through autoconditioning prior to isolation (Parsana et al., 2012), which is potentially facilitated by biological preparedness (Wöhr and Schwarting, 2010).

Most importantly, however, our findings demonstrate for the first time that post-weaning isolation not only induces behavioral changes displayed by the sender, but also by the receiver in response to 50-kHz USV. This is in line with a study on scent marking behavior where post-weaning long-term isolation increased the time spent investigating conspecific odors, but reduced the amount of urine counter-marking, together with an alteration in odor preferences (Brown, 1991). Lack of approach behavior during 50-kHz USV playback following long-term isolation could be interpreted in terms of increased anxiety and/or reduced social motivation, possibly resembling a depression-like behavioral phenotype (Wright et al., 1991; van den Berg et al., 1999b; Lukkes et al., 2009; Kokare et al., 2010; Meng et al., 2010). The idea that social isolation affects the level of social motivation in the receiver is supported by our bidirectional modulation of approach behavior in response to 50-kHz USV through short- and long-term isolation. While group-housed rats showed a clear preference toward 50-kHz USV, this response was enhanced after short-term isolation, whereas it was absent in long-term isolated rats. In fact, there is evidence that increased social motivation in the sender is reflected by higher rates of 50-kHz USV emission, e.g., during social play or tickling (Knutson et al., 1998; Panksepp and Burgdorf, 2000; Burgdorf and Panksepp, 2001).

A further explanation for the lack of approach behavior following post-weaning long-term isolation, is a deficit in socio-affective information processing, induced by withholding social play. Importantly, play behavior reaches its peak during the middle of the juvenile stage (Panksepp, 1981; Thor and Holloway, 1984), exactly during the period of isolation. Play fighting differs from serious fighting in regards to the target of attack (Takahashi and Lore, 1983; Pellis and Pellis, 1987), and consists of various behavioral components which appear to be programmed during development, promoting reciprocity, i.e., comparable outcome of successful play encounters within a pair, by reducing the behavioral control over the partner (Pellis, 2002; Pellis et al., 2005). It is assumed that social play facilitates neural and behavioral development by equipping an individual adequately for the needs in adulthood (Vanderschuren et al., 1997). In support of the critical period hypothesis (Scott, 1962; Hol et al., 1999; van den Berg et al., 1999a), a full manifestation of isolation-induced behavioral alterations is only observed when the intervention takes place during this sensitive developmental period (Fone and Porkess, 2008). During play, juveniles frequently experience a loss of control by exposing themselves to unpredictable events. Training for the unexpected allows rats to develop flexible responses to cope with rapidly changing environments (Spinka et al., 2001; Pellis et al., 2010). Most notably, physical interactions during adolescence are important to acquire social competency. This includes social, emotional, and cognitive skills; such as the ability to express and understand communicative signals from conspecifics (Wöhr and Schwarting, 2013).

While deficits in approach behavior were induced by post-weaning isolation, no such effects were seen in young adults reared in post-adolescent isolation, i.e., after going through the rough-and-tumble play period. This is also in line with other studies that could not find behavioral deficits in rats that were at least partially reared in groups during this critical period, before being isolated (Wright et al., 1991; Hol et al., 1999). There is some evidence that additional playful experience during the course of isolation may, to some extent, prevent impaired development of social competencies (Potegal and Einon, 1989; Einon and Potegal, 1991; but see van den Berg et al., 2000), further suggesting that there is a critical involvement of play behavior on socio-affective information processing in rats.

In support of this notion, it was shown in a well-established autism model that social deficits can be improved by peer intervention (Yang et al., 2011). In our study, deficits in approach induced by post-weaning isolation were partially rescued by 1 week of group housing. Yet, other studies investigating behavioral changes following re-socialization are inconsistent. For instance, it was shown that increased anxiety, abnormal aggression, and defense behavior, as well as reduced social interaction were not alleviated by re-socialization (Potegal and Einon, 1989; Einon and Potegal, 1991; Wright et al., 1991; van den Berg et al., 1999a; Lukkes et al., 2009; Tulogdi et al., 2014). In line with our results, however, beneficial effects of re-socialization could be demonstrated, like attenuation of anxiety-like behavior, depression-like behavior, and isolation-induced aggression (Vale and Montgomery, 1997; Kokare et al., 2010; Meng et al., 2010), as well as recovery of sleep-related huddling (Tulogdi et al., 2014) and social interaction (Kokare et al., 2010). There is also evidence for restored social cooperation by re-socialization after isolation during adulthood (Willner et al., 1989). Data from human adoption studies further support a re-socialization effect insofar as placement of children in a beneficial family environment was shown to reverse deficits in cognitive development, as compared to children remaining in institutional care (Nelson et al., 2007; Bos et al., 2011).

Apparently, efficacy of rat re-socialization depends both on isolation duration (Hol et al., 1999) and re-socialization duration, with at least 3 days of social housing being required to attenuate certain behavioral deficits (Kokare et al., 2010). Regarding our results, 1 week of group housing might still not be sufficient to completely rescue the adverse effects caused by 4 weeks of post-weaning isolation in this paradigm. Also, it appears possible that social experiences made by the peers before peer intervention play an important role (Hol et al., 1999; Tulogdi et al., 2014). In the future it would therefore be interesting to assess the effects of longer peer intervention periods and previous social experiences made by the peers.

Importantly, behavioral alterations following post-weaning isolation, such as deficits in the social behavior repertoire, mimic negative symptoms typically observed in schizophrenia (Hall, 1998; Lapiz et al., 2003; Fone and Porkess, 2008). Our present finding that post-weaning long-term isolation results in a complete lack of approach behavior in the receiver when exposed to 50-kHz USV is in line with that view. In fact, in schizophrenia patients, several domains of social behavior are impaired (Penn et al., 2008), including theory of mind (Brüne, 2005; Biedermann et al., 2012), emotional perception (Schneider et al., 2006; Irani et al., 2012), and verbal and non-verbal communication (Couture et al., 2006; Lavelle et al., 2013). According to the DSM-5 (American Psychiatric Association, 2013), patients display severe disorganized and/or reduced speech output (alogia) that substantially impairs effective communication, as well as diminished emotional expression and a lack of interest in social interactions (asociality).

Consistently, pharmacological treatments, including antipsychotics, were shown to rescue isolation-induced deficits in rats. Sub-chronic, but not acute administration of the antipsychotic drug clozapine or N-acetyl cysteine, a modulator of glutamatergic transmission, restores social interaction and deficits in other domains linked to schizophrenia, such as sensorimotor gating and memory (Möller et al., 2011, 2013). Also, acute treatment with the selective MC4 receptor antagonist HS014 reverses social deficits, as well as anxiety- and depression-like symptoms (Kokare et al., 2010). In contrast, benzodiazepines attenuate increased aggression but the effects on social interaction are inconsistent, thus challenging the notion that isolation-induced abnormal social behavior is anxiety-related (Wongwitdecha and Marsden, 1996; Vale and Montgomery, 1997). Furthermore, chronic application of the antidepressants imipramine or fluoxetine, after isolation during adulthood, was demonstrated to re-establish cooperative behavior (Willner et al., 1989). Importantly, chronic morphine treatment counteracts reduced social activity, accompanied by a reversal of upregulated μ-opioid-receptor binding sites (van den Berg et al., 1999c, 2000). Since it was shown that social play induces the release of opioids in specific brain regions (Vanderschuren et al., 1997), alterations in social behavior due to deprivation of play might be the result of reduced opioid release during this critical developmental period. Morphine treatment appears to substitute for the lack of play-induced opioid peptide release. Consistently, knocking-out μ-opioid-receptors in mice (Wöhr et al., 2011) or administering a μ-opioid-receptor-antagonist in rats (Wöhr and Schwarting, 2009) negatively affects approach behavior during USV playback, suggesting that endogenous opioids are not only involved in social behavior but also in socio-affective information processing (Oddi et al., 2013).

Besides the systems targeted in these pharmacological studies, behavioral alterations observed following post-weaning isolation are further accompanied by various neuromorphological changes and neurochemical imbalances, including neurotransmitter systems implicated in schizophrenia, such as dopamine and serotonin (Hall, 1998; Lapiz et al., 2003; Fone and Porkess, 2008). Consistently, by means of the post-weaning isolation paradigm applied here, we recently induced alterations in regulators of neuronal development and synaptic plasticity, such as ubiquitin ligase and microRNAs (Valluy et al., 2015). In future studies, it would therefore be interesting to assess changes in neurotransmission and synaptic plasticity that specifically occur in response to post-weaning, but not post-adolescent isolation, and to test which of them can be reversed by peer intervention. This approach would likely lead to the identification of promising targets for novel pharmacological treatment approaches, the efficacy of which could be tested in rodent models for negative symptoms of schizophrenia.

## Conclusion

We showed that post-weaning social isolation specifically affected the behavioral response to playback of pro-social 50-kHz but not alarm 22-kHz USV. While group-housed rats showed the expected preference toward 50-kHz USV, the response was even stronger in short-term isolated rats, possibly due to a higher level of social motivation. In contrast, no approach behavior to 50-kHz USV was observed in long-term isolated rats. Importantly, deficits in approach behavior were reversed by peer-mediated re-socialization and could not be observed after post-adolescent social isolation, indicating a critical period for social development during adolescence. Together, these results highlight the importance of social experience for affiliative behavior, suggesting a critical involvement of play behavior on socio-affective information processing in rats.

### Conflict of interest statement

The authors declare that the research was conducted in the absence of any commercial or financial relationships that could be construed as a potential conflict of interest.

## References

[B1] American Psychiatric Association. (2013). Diagnostic and Statistical Manual of Mental Disorders, 5th Edn. Arlington, VA: American Psychiatric Publishing 10.1176/appi.books.9780890425596

[B2] Baenninger L. P. (1966). The reliability of dominance orders in rats. Anim. Behav. 14, 367–371. 10.1016/S0003-3472(66)80099-4 6006026

[B3] Ben-Ami Bartal I. Decety J. Mason P. (2011). Empathy and pro-social behavior in rats. Science 334, 1427–1430. 10.1126/science.1210789 22158823 PMC3760221

[B4] Biedermann F. Frajo-Apor B. Hofer A. (2012). Theory of mind and its relevance in schizophrenia. Curr. Opin. Psychiatr. 25, 71–75. 10.1097/YCO.0b013e3283503624 22249083

[B5] Borg E. (1982). Auditory thresholds in rats of different age and strain. A behavioral and electrophysiological study. Hear. Res. 8, 101–115. 10.1016/0378-5955(82)90069-7 7142038

[B6] Bos K. Zeanah C. H. Fox N. A. Drury S. S. McLaughlin K. A. Nelson C. A. III. (2011). Psychiatric outcomes in young children with a history of institutionalization. Harv. Rev. Psychiatry 19, 15–24. 10.3109/10673229.2011.549773 21250893 PMC3445019

[B7] Braun K. Bock J. (2011). The experience-dependent maturation of prefronto-limbic circuits and the origin of developmental psychopathology: implications for the pathogenesis and therapy of behavioural disorders. Dev. Med. Child Neurol. 53, 14–18. 10.1111/j.1469-8749.2011.04056.x 21950388

[B8] Brown J. Cohen P. Johnson J. G. Smailes E. M. (1999). Childhood abuse and neglect: specificity of effects on adolescent and young adult depression and suicidality. J. Am. Acad. Child Adolesc. Psychiatry 38, 1490–1496. 10.1097/00004583-199912000-00009 10596248

[B9] Brown R. E. (1991). Effects of rearing condition, gender, and sexual experience on odor preferences and urine marking in Long-Evans rats. Anim. Learn. Behav. 19, 18–28 10.3758/BF03197856

[B10] Brudzynski S. M. (2013). Ethotransmission: communication of emotional states through ultrasonic vocalization in rats. Curr. Opin. Neurobiol. 23, 310–317. 10.1016/j.conb.2013.01.014 23375168

[B11] Brüne M. (2005). “Theory of mind” in schizophrenia: a review of the literature. Schizophr. Bull. 31, 21–42. 10.1093/schbul/sbi002 15888423

[B12] Burgdorf J. Panksepp J. (2001). Tickling induces reward in adolescent rats. Physiol. Behav. 72, 167–173. 10.1016/S0031-9384(00)00411-X 11239994

[B13] Burman O. H. P. Ilyat A. Jones G. Mendl M. (2007). Ultrasonic vocalizations as indicators of welfare for laboratory rats (*Rattus norvegicus*). Appl. Anim. Behav. Sci. 104, 116–129. 10.1016/j.applanim.2006.04.02824602543

[B14] Cicchetti D. Toth S. L. (1995). A developmental psychopathology perspective on child abuse and neglect. J. Am. Acad. Child Adolesc. Psychiatry 34, 541–565. 10.1097/00004583-199505000-00008 7775351

[B15] Couture S. M. Penn D. L. Roberts D. L. (2006). The functional significance of social cognition in schizophrenia: a review. Schizophr. Bull. 32(Suppl. 1), S44–63. 10.1093/schbul/sbl029 16916889 PMC2632537

[B16] DeRosse P. Nitzburg G. C. Kompancaril B. Malhotra A. K. (2014). The relation between childhood maltreatment and psychosis in patients with schizophrenia and non-psychiatric controls. Schizophr. Res. 155, 66–71. 10.1016/j.schres.2014.03.009 24704218 PMC4050634

[B17] Dodge K. A. Bates J. E. Pettit G. S. (1990). Mechanisms in the cycle of violence. Science 250, 1678–1683. 10.1126/science.2270481 2270481

[B18] Einon D. Potegal M. (1991). Enhanced defense in adult rats deprived of playfighting experience as juveniles. Aggress. Behav. 17, 27–40. 2925003 10.1002/dev.420220206

[B19] Endres T. Widmann K. Fendt M. (2007). Are rats predisposed to learn 22 kHz calls as danger-predicting signals? Behav. Brain Res. 185, 69–75. 10.1016/j.bbr.2007.07.012 17714801

[B20] Ferdman N. Murmu R. P. Bock J. Braun K. Leshem M. (2007). Weaning age, social isolation, and gender, interact to determine adult explorative and social behavior, and dendritic and spine morphology in prefrontal cortex of rats. Behav. Brain Res. 180, 174–182. 10.1016/j.bbr.2007.03.011 17477981

[B21] Fone K. C. F. Porkess M. V. (2008). Behavioural and neurochemical effects of post-weaning social isolation in rodents—relevance to developmental neuropsychiatric disorders. Neurosci. Biobehav. Rev. 32, 1087–1102. 10.1016/j.neubiorev.2008.03.003 18423591

[B22] Frank D. A. Klass P. E. Earls F. Eisenberg L. (1996). Infants and young children in orphanages: one view from pediatrics and child psychiatry. Pediatrics 97, 569–578. 8632947

[B23] Gaskin P. L. R. Alexander S. P. H. Fone K. C. F. (2014). Neonatal phencyclidine administration and post-weaning social isolation as a dual-hit model of ‘schizophrenia-like’ behaviour in the rat. Psychopharmacology 231, 2533–2545. 10.1007/s00213-013-3424-y 24402141

[B24] Gilbert R. Widom C. S. Browne K. Fergusson D. Webb E. Janson S. (2009). Burden and consequences of child maltreatment in high-income countries. Lancet 373, 68–81. 10.1016/S0140-6736(08)61706-7 19056114

[B25] Grotewold S. K. Wall V. L. Goodell D. J. Hayter C. Bland S. T. (2014). Effects of cocaine combined with a social cue on conditioned place preference and nucleus accumbens monoamines after isolation rearing in rats. Psychopharmacology 231, 3041–3053. 10.1007/s00213-014-3470-0 24553577 PMC4646085

[B26] Hall F. S. (1998). Social deprivation of neonatal, adolescent, and adult rats has distinct neurochemical and behavioral consequences. Crit. Rev. Neurobiol. 12, 129–162. 10.1615/CritRevNeurobiol.v12.i1-2.50 9444483

[B27] Han X. Wang W. Shao F. Li N. (2011). Isolation rearing alters social behaviors and monoamine neurotransmission in the medial prefrontal cortex and nucleus accumbens of adult rats. Brain Res. 1385, 175–181. 10.1016/j.brainres.2011.02.035 21338587

[B28] Hernandez-Lallement J. van Wingerden M. Marx C. Srejic M. Kalenscher T. (2015). Rats prefer mutual rewards in a prosocial choice task. Front. Neurosci. 8:443. 10.3389/fnins.2014.00443 25642162 PMC4296215

[B29] Himmler B. T. Kisko T. M. Euston D. R. Kolb B. Pellis S. M. (2014). Are 50-kHz calls used as play signals in the playful interactions of rats? I. Evidence from the timing and context of their use. Behav. Process. 106, 60–66. 10.1016/j.beproc.2014.04.014 24811452

[B30] Hol T. van den Berg C. L. van Ree J. M. Spruijt B. M. (1999). Isolation during the play period in infancy decreases adult social interactions in rats. Behav. Brain Res. 100, 91–97. 10.1016/S0166-4328(98)00116-8 10212056

[B31] Inagaki H. Kuwahara M. Tsubone H. Mori Y. (2013). The effect of post-weaning individual housing on 50-kHz calls emitted from male rats to sexually receptive female rats. Physiol. Behav. 110–111, 30–33. 10.1016/j.physbeh.2012.11.009 23220361

[B32] Irani F. Seligman S. Kamath V. Kohler C. Gur R. C. (2012). A meta-analysis of emotion perception and functional outcomes in schizophrenia. Schizophr. Res. 137, 203–211. 10.1016/j.schres.2012.01.023 22341200 PMC3351501

[B33] Kisko T. M. Himmler B. T. Himmler S. M. Euston D. R. Pellis S. M. (2015). Are 50-kHz calls used as play signals in the playful interactions of rats? II. Evidence from the effects of devocalization. Behav. Process. 111, 25–33. 10.1016/j.beproc.2014.11.011 25447515

[B34] Knutson B. Burgdorf J. Panksepp J. (1998). Anticipation of play elicits high-frequency ultrasonic vocalizations in young rats. J. Comp. Psychol. 112, 65–73. 10.1037/0735-7036.112.1.65 9528115

[B35] Kokare D. M. Dandekar M. P. Singru P. S. Gupta G. L. Subhedar N. K. (2010). Involvement of α-MSH in the social isolation induced anxiety- and depression-like behaviors in rat. Neuropharmacology 58, 1009–1018. 10.1016/j.neuropharm.2010.01.006 20080115

[B36] Lansford J. E. Dodge K. A. Pettit G. S. Bates J. E. Crozier J. Kaplow J. (2002). A 12-year prospective study of the long-term effects of early child physical maltreatment on psychological, behavioral, and academic problems in adolescence. Arch. Pediatr. Adolesc. Med. 156, 824–830. 10.1001/archpedi.156.8.824 12144375 PMC2756659

[B37] Lapiz M. D. S. Fulford A. Muchimapura S. Mason R. Parker T. Marsden C. A. (2003). Influence of postweaning social isolation in the rat on brain development, conditioned behavior, and neurotransmission. Neurosci. Behav. Physiol. 33, 13–29. 10.1023/A:1021171129766 12617300

[B38] Lavelle M. Healey P. G. T. McCabe R. (2013). Is nonverbal communication disrupted in interactions involving patients with schizophrenia? Schizophr. Bull. 39, 1150–1158. 10.1093/schbul/sbs091 22941744 PMC3756773

[B39] Łopuch S. Popik P. (2011). Cooperative behavior of laboratory rats (*Rattus norvegicus*) in an instrumental task. J. Comp. Psychol. 125, 250–253. 10.1037/a0021532 21341907

[B40] Lukkes J. L. Mokin M. V. Scholl J. L. Forster G. L. (2009). Adult rats exposed to early-life social isolation exhibit increased anxiety and conditioned fear behavior, and altered hormonal stress responses. Horm. Behav. 55, 248–256. 10.1016/j.yhbeh.2008.10.014 19027017

[B41] Meaney M. J. Stewart J. (1979). Environmental factors influencing the affiliative behavior of male and female rats (*Rattus norvegicus*). Anim. Learn. Behav. 7, 397–405. 10.3758/BF0320969220363720

[B42] Meng Q. Li N. Han X. Shao F. Wang W. (2010). Peri-adolescence isolation rearing alters social behavior and nociception in rats. Neurosci. Lett. 480, 25–29. 10.1016/j.neulet.2010.05.067 20638959

[B43] Möller M. Du Preez J. L. Emsley R. Harvey B. H. (2011). Isolation rearing-induced deficits in sensorimotor gating and social interaction in rats are related to cortico-striatal oxidative stress, and reversed by sub-chronic clozapine administration. Eur. Neuropsychopharm. 21, 471–483 10.1016/j.euroneuro.2010.09.006 20965701

[B44] Möller M. Du Preez J. L. Viljoen F. P. Berk M. Emsley R. Harvey B. H. (2013). Social isolation rearing induces mitochondrial, immunological, neurochemical and behavioural deficits in rats, and is reversed by clozapine or N-acetyl cysteine. Brain Behav. Immun. 30, 156–167. 10.1016/j.bbi.2012.12.011 23270677

[B45] Nelson C. A. III Zeanah C. H. Fox N. A. Marshall P. J. Smyke A. T. Guthrie D. (2007). Cognitive recovery in socially deprived young children: the Bucharest Early Intervention Project. Science 318, 1937–1940. 10.1126/science.1143921 18096809

[B46] Oddi D. Crusio W. E. D'Amato F. R. Pietropaolo S. (2013). Monogenic mouse models of social dysfunction: implications for autism. Behav. Brain Res. 251, 75–84. 10.1016/j.bbr.2013.01.002 23327738

[B47] Panksepp J. (1981). The ontogeny of play in rats. Dev. Psychobiol. 14, 327–332. 10.1002/dev.420140405 7250521

[B48] Panksepp J. Burgdorf J. (2000). 50-kHz chirping (laughter?) in response to conditioned and unconditioned tickle-induced reward in rats: effects of social housing and genetic variables. Behav. Brain Res. 115, 25–38. 10.1016/S0166-4328(00)00238-2 10996405

[B49] Parsana A. J. Moran E. E. Brown T. H. (2012). Rats learn to freeze to 22-kHz ultrasonic vocalizations through autoconditioning. Behav. Brain Res. 232, 395–399. 10.1016/j.bbr.2012.03.031 22475554 PMC3367110

[B50] Pellis S. M. (2002). Keeping in touch: Play fighting and social knowledge, in The Cognitive Animal. Empirical and Theoretical Perspectives on Animal Cognition, eds Bekoff M. Allen C. Burghardt G. M. (Cambridge, MA: MIT Press), 421–427.

[B51] Pellis S. M. Pellis V. C. (1987). Play-fighting differs from serious fighting in both target of attack and tactics of fighting in the laboratory rat *Rattus norvegicus*. Aggress. Behav. 13, 227–242.

[B52] Pellis S. M. Pellis V. C. Bell H. C. (2010). The function of play in the development of the social brain. Am. J. Play 2, 278–296.

[B53] Pellis S. M. Pellis V. C. Foroud A. (2005). Play fighting: Aggression, affiliation, and the development of nuanced social skills, in Developmental Origins of Aggression, eds Tremblay R. E. Hartup W. W. Archer J. (New York, NY: Guilford Press), 47–62.

[B54] Penn D. L. Sanna L. J. Roberts D. L. (2008). Social cognition in schizophrenia: an overview. Schizophr. Bull. 34, 408–411. 10.1093/schbul/sbn014 18375928 PMC2632430

[B55] Portfors C. V. (2007). Types and functions of ultrasonic vocalizations in laboratory rats and mice. J. Am. Assoc. Lab. Anim. Sci. 46, 28–34. 17203913

[B56] Potegal M. Einon D. (1989). Aggressive behaviors in adult rats deprived of playfighting experience as juveniles. Dev. Psychobiol. 22, 159–172. 10.1002/dev.420220206 2925003

[B57] Read J. Os J. Morrison A. P. Ross C. A. (2005). Childhood trauma, psychosis and schizophrenia: a literature review with theoretical and clinical implications. Acta Psychiatr. Scand. 112, 330–350. 10.1111/j.1600-0447.2005.00634.x 16223421

[B58] Rutte C. Taborsky M. (2007). Generalized reciprocity in rats. PLoS Biol. 5:e196. 10.1371/journal.pbio.0050196 17608566 PMC1914408

[B59] Rutte C. Taborsky M. (2008). The influence of social experience on cooperative behaviour of rats (*Rattus norvegicus*): direct vs generalised reciprocity. Behav. Ecol. Sociobiol. 62, 499–505 10.1007/s00265-007-0474-3

[B60] Sales G. D. (1972). Ultrasound and aggressive behaviour in rats and other small mammals. Anim. Behav. 20, 88–100. 10.1016/S0003-3472(72)80177-5 4677167

[B61] Schneider F. Gur R. C. Koch K. Backes V. Amunts K. Shah N. J.. (2006). Impairment in the specificity of emotion processing in schizophrenia. Am. J. Psychiatry 163, 442–447. 10.1176/appi.ajp.163.3.442 16513865

[B62] Scott J. P. (1962). Critical periods in behavioral development. Science 138, 949–958. 10.1126/science.138.3544.949 13992547

[B63] Seffer D. Schwarting R. K. W. Wöhr M. (2014). Pro-social ultrasonic communication in rats: insights from playback studies. J. Neurosci. Methods 234, 73–81. 10.1016/j.jneumeth.2014.01.023 24508146

[B64] Shackman J. E. Pollak S. D. (2014). Impact of physical maltreatment on the regulation of negative affect and aggression. Dev. Psychopathol. 26, 1021–1033. 10.1017/S0954579414000546 24914736 PMC4608022

[B65] Silberberg A. Allouch C. Sandfort S. Kearns D. Karpel H. Slotnick B. (2014). Desire for social contact, not empathy, may explain “rescue” behavior in rats. Anim. Cogn. 17, 609–618. 10.1007/s10071-013-0692-1 24126919

[B66] Spinhoven P. Elzinga B. M. Hovens J. G. F. M. Roelofs K. Zitman F. G. van Oppen P. (2010). The specificity of childhood adversities and negative life events across the life span to anxiety and depressive disorders. J. Affect. Disorders 126, 103–112 10.1016/j.jad.2010.02.132 20304501

[B67] Spinka M. Newberry R. C. Bekoff M. (2001). Mammalian play: training for the unexpected. Q. Rev. Biol. 76, 141–168. 10.1086/393866 11409050

[B68] Takahashi L. K. Lore R. K. (1983). Play fighting and the development of agonistic behavior in male and female rats. Aggress. Behav. 9, 217–227.

[B69] Thor D. H. Holloway W. R. Jr. (1984). Developmental analyses of social play behavior in juvenile rats. Bull. Psychon. Soc. 22, 587–590 10.3758/BF03333916

[B70] Tóth M. Halász J. Mikics É. Barsy B. Haller J. (2008). Early social deprivation induces disturbed social communication and violent aggression in adulthood. Behav. Neurosci. 122, 849–854. 10.1037/0735-7044.122.4.849 18729638

[B71] Tulogdi Á. Tóth M. Barsvári B. Biró L. Mikics É. Haller J. (2014). Effects of resocialization on post-weaning social isolation-induced abnormal aggression and social deficits in rats. Dev. Psychobiol. 56, 49–57. 10.1002/dev.21090 23168609

[B72] Vale A. L. Montgomery A. M. (1997). Social interaction: responses to chlordiazepoxide and the loss of isolation-reared effects with paired-housing. Psychopharmacology 133, 127–132. 10.1007/s002130050382 9342778

[B73] Valluy J. Bicker S. Aksoy-Aksel A. Lackinger M. Sumer S. Fiore R. (2015). A coding-independent function of an alternative Ube3a transcript during neuronal development. Nat. Neurosci. [Epub ahead of print]. 10.1038/nn.3996. 25867122

[B75] van den Berg C. L. Hol T. van Ree J. M. Spruijt B. M. Everts H. Koolhaas J. M. (1999a). Play is indispensable for an adequate development of coping with social challenges in the rat. Dev. Psychobiol. 34, 129–138. 10086231

[B77] van den Berg C. L. Pijlman F. T. A. Koning H. A. M. Diergaarde L. van Ree J. M.. (1999b). Isolation changes the incentive value of sucrose and social behaviour in juvenile and adult rats. Behav. Brain Res. 106, 133–142. 10595429 10.1016/s0166-4328(99)00099-6

[B76] van den Berg C. L. van Ree J. M. Spruijt B. M. (2000). Morphine attenuates the effects of juvenile isolation in rats. Neuropharmacology 39, 969–976. 10.1016/S0028-3908(99)00216-6 10727707

[B74] van den Berg C. L. van Ree J. M. Spruijt B. M. Kitchen I. (1999c). Effects of juvenile isolation and morphine treatment on social interactions and opioid receptors in adult rats: behavioural and autoradiographic studies. Eur. J. Neurosci. 11, 3023–3032. 10510167 10.1046/j.1460-9568.1999.00717.x

[B78] Vanderschuren L. J. M. J. Niesink R. J. M. van Ree J. M. (1997). The neurobiology of social play behavior in rats. Neurosci. Biobehav. Rev. 21, 309–326. 10.1016/S0149-7634(96)00020-6 9168267

[B79] Varese F. Smeets F. Drukker M. Lieverse R. Lataster T. Viechtbauer W.. (2012). Childhood adversities increase the risk of psychosis: a meta-analysis of patient-control, prospective- and cross-sectional cohort studies. Schizophr. Bull. 38, 661–671. 10.1093/schbul/sbs050 22461484 PMC3406538

[B80] Veenema A. H. (2009). Early life stress, the development of aggression and neuroendocrine and neurobiological correlates: what can we learn from animal models? Front. Neuroendocrinol. 30, 497–518. 10.1016/j.yfrne.2009.03.003 19341763

[B81] von Frijtag J. C. Schot M. van den Bos R. Spruijt B. M. (2002). Individual housing during the play period results in changed responses to and consequences of a psychosocial stress situation in rats. Dev. Psychobiol. 41, 58–69. 10.1002/dev.10057 12115291

[B82] Wall V. L. Fischer E. K. Bland S. T. (2012). Isolation rearing attenuates social interaction-induced expression of immediate early gene protein products in the medial prefrontal cortex of male and female rats. Physiol. Behav. 107, 440–450. 10.1016/j.physbeh.2012.09.002 22982514 PMC4529065

[B83] Whishaw I. Q. Kolb B. (2005). The Behavior of the Laboratory Rat: A Handbook with Tests. New York, NY: Oxford University Press.

[B84] Willner P. Sampson D. Phillips G. Fichera R. Foxlow P. Muscat R. (1989). Effects of isolated housing and chronic antidepressant treatment on cooperative social behaviour in rats. Behav. Pharmacol. 1, 85–90. 10.1097/00008877-198900110-00010 11175390

[B85] Willuhn I. Tose A. Wanat M. J. Hart A. S. Hollon N. G. Phillips P. E. M.. (2014). Phasic dopamine release in the nucleus accumbens in response to pro-social 50 kHz ultrasonic vocalizations in rats. J. Neurosci. 34, 10616–10623. 10.1523/JNEUROSCI.1060-14.2014 25100595 PMC4200110

[B86] Wöhr M. Borta A. Schwarting R. K. W. (2005). Overt behavior and ultrasonic vocalization in a fear conditioning paradigm: a dose–response study in the rat. Neurobiol. Learn. Mem. 84, 228–240. 10.1016/j.nlm.2005.07.004 16115784

[B87] Wöhr M. Houx B. Schwarting R. K. W. Spruijt B. (2008). Effects of experience and context on 50-kHz vocalizations in rats. Physiol. Behav. 93, 766–776. 10.1016/j.physbeh.2007.11.031 18191963

[B88] Wöhr M. Moles A. Schwarting R. K. W. D'Amato F. R. (2011). Lack of social exploratory activation in male μ-opioid receptor KO mice in response to playback of female ultrasonic vocalizations. Soc. Neurosci. 6, 76–87. 10.1080/17470911003765560 20503133

[B89] Wöhr M. Schwarting R. K. W. (2007). Ultrasonic communication in rats: can playback of 50-kHz calls induce approach behavior? PLoS ONE 2:e1365. 10.1371/journal.pone.0001365 18159248 PMC2137933

[B90] Wöhr M. Schwarting R. K. W. (2009). Ultrasonic communication in rats: effects of morphine and naloxone on vocal and behavioral responses to playback of 50-kHz vocalizations. Pharmacol. Biochem. Behav. 94, 285–295. 10.1016/j.pbb.2009.09.008 19758572

[B91] Wöhr M. Schwarting R. K. W. (2010). Activation of limbic system structures by replay of ultrasonic vocalization in rats, in Handbook of Mammalian Vocalization. An Integrative Neuroscience Approach, ed Brudzynski S. (Amsterdam: Elsevier - Academic Press), 113–124 10.1016/B978-0-12-374593-4.00012-7

[B92] Wöhr M. Schwarting R. K. W. (2012). Testing social acoustic memory in rats: effects of stimulus configuration and long-term memory on the induction of social approach behavior by appetitive 50-kHz ultrasonic vocalizations. Neurobiol. Learn. Mem. 98, 154–164. 10.1016/j.nlm.2012.05.004 22677211

[B93] Wöhr M. Schwarting R. K. W. (2013). Affective communication in rodents: ultrasonic vocalizations as a tool for research on emotion and motivation. Cell Tissue Res. 354, 81–97. 10.1007/s00441-013-1607-9 23576070

[B94] Wongwitdecha N. Marsden C. A. (1996). Social isolation increases aggressive behaviour and alters the effects of diazepam in the rat social interaction test. Behav. Brain Res. 75, 27–32. 10.1016/0166-4328(96)00181-7 8800657

[B95] Wright I. K. Upton N. Marsden C. A. (1991). Resocialisation of isolation-reared rats does not alter their anxiogenic profile on the elevated X-maze model of anxiety. Physiol. Behav. 50, 1129–1132. 10.1016/0031-9384(91)90572-6 1798767

[B96] Yang M. Perry K. Weber M. D. Katz A. M. Crawley J. N. (2011). Social peers rescue autism-relevant sociability deficits in adolescent mice. Autism Res. 4, 17–27. 10.1002/aur.163 20928844 PMC3065860

