# Supplementary material for: Pro-social 50-kHz ultrasonic communication in rats: post-weaning but not post-adolescent social isolation leads to social impairments—phenotypic rescue by re-socialization
Source: Front Behav Neurosci. 2015 May 1;9:102. doi: 10.3389/fnbeh.2015.00102 (PMC4416445; doi:10.3389/fnbeh.2015.00102)
Supplement: Supplementary file 1 [file DataSheet1.DOCX]

***Supplementary Material***

**Pro-social 50-kHz ultrasonic communication in rats: Post-weaning but not post-adolescent social isolation leads to social impairments – phenotypic rescue by re-socialization**

Short title: Post-weaning isolation induces communication deficits

*Original Research Article*

**Dominik Seffer, Henrike Rippberger, Rainer K.W. Schwarting & Markus Wöhr**

Behavioral Neuroscience, Experimental and Biological Psychology,

Philipps-University of Marburg, Marburg, Germany

Correspondence should be addressed to:

Dominik Seffer, Dipl.Biol.

Behavioral Neuroscience

Faculty of Psychology

Philipps-University of Marburg

Gutenbergstr. 18, 35032 Marburg,

Germany

Tel: +496421 2823646

Fax: +496421 2823610

e-mail: seffer@staff.uni-marburg.de

Or to:

Markus Wöhr, PhD

Behavioral Neuroscience

Faculty of Psychology

Philipps-University of Marburg

Gutenbergstr. 18, 35032 Marburg,

Germany

Tel: +496421 2823612

Fax: +496421 2823610

e-mail: woehrm@staff.uni-marburg.de

**SUPPLEMENTARY FIGURES**

**
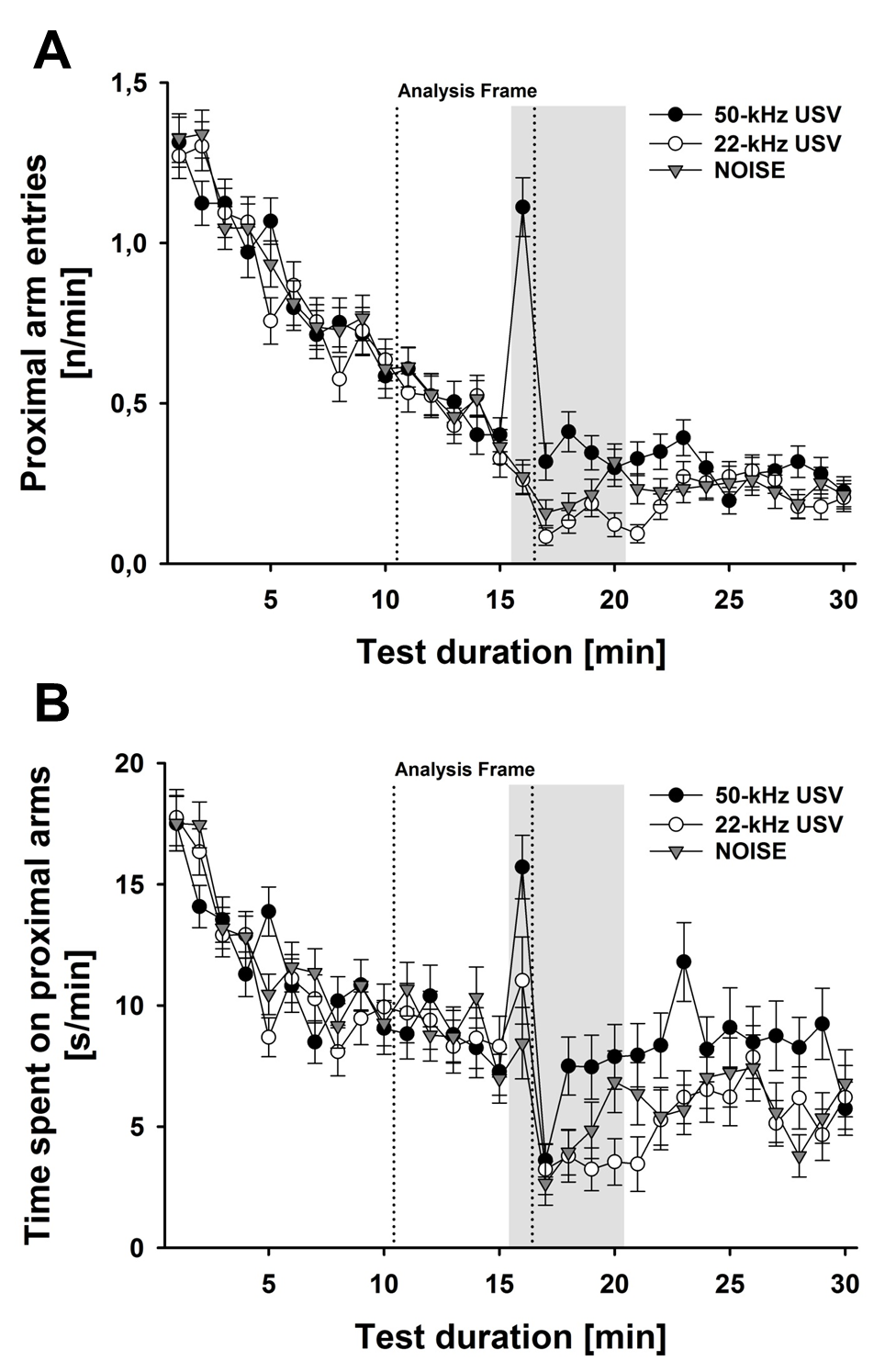
**

**Supplementary Figure 1: Social approach behavior is specifically induced by pro-social 50-kHz USV but not alarm 22-kHz USV and NOISE. (A)** Line graphs depicting the time course of proximal arm entries [n/min] displayed by rats in response to 50-kHz USV (black circles), 22-kHz USV (white circles), and NOISE (grey triangles), irrespective of experimental housing condition in Experiment 1: Post-weaning Social Isolation - Effects. **(B)** Line graphs depicting the time course of the time spent on proximal arms [s/min] displayed by rats in response to 50-kHz USV (black circles), 22-kHz USV (white circles), and NOISE (grey triangles), irrespective of experimental housing condition in Experiment 1: Post-weaning Social Isolation - Effects. The playback phase is highlighted in grey. The dotted lines represent the analysis frame used in all three experiments: Experiment 1: Post-weaning Social Isolation – Effects; Experiment 2: Post-weaning Social Isolation – Rescue; and Experiment 3: Post-adolescent Social Isolation - Effects.


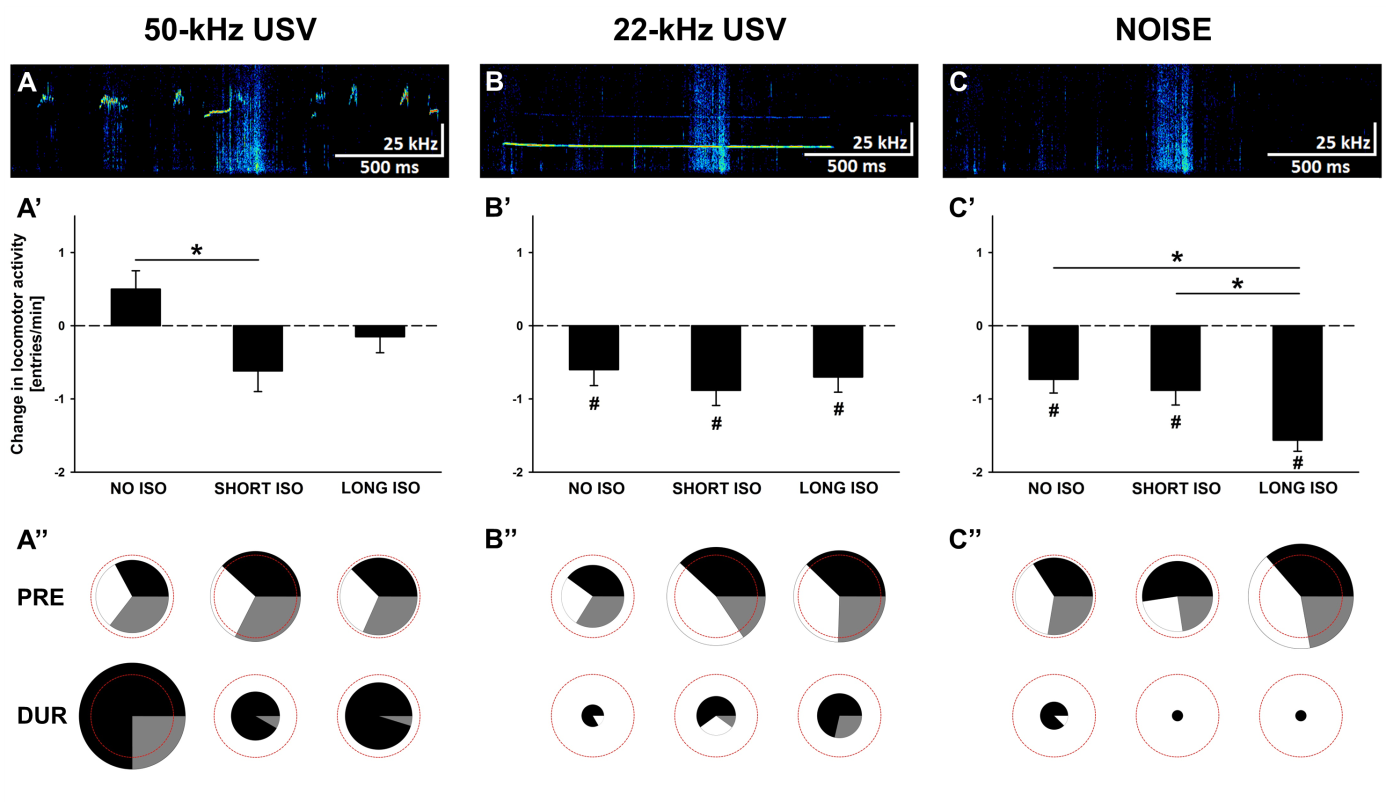


**Supplementary Figure 2: Post-weaning social isolation induced behavioral inhibition in response to pro-social 50-kHz USV similar to alarm 22-kHz USV and NOISE can be reversed by re-socialization.** **(A)**, **(B)**, and **(C)** Exemplary spectrograms of acoustic stimuli used for playback, namely **(A)** 50-kHz USV, **(B)** 22-kHz USV, and **(C)**, background noise (NOISE). **(A’)**, **(B’)**, and **(C’)** Bar graphs depicting changes in locomotor activity as assessed by means of total arm entries per min [entries/min] displayed by rats exposed to four weeks of NO ISO (left), SHORT ISO (middle), and LONG ISO (right), post-weaning, plus one additional week of peer rearing, in response to **(A’)** 50-kHz USV, **(B’)** 22-kHz USV, and **(C’)** NOISE, in Experiment 2: Post-weaning Social Isolation - Rescue. **(A’’)**, **(B’’)**, and **(C’’)**, Pie charts depicting changes in stimulus-directed locomotor activity as assessed by means of numbers of proximal (black), neutral (grey), and distal (white) arm entries displayed as percentages by rats exposed to four weeks of NO ISO (left), SHORT ISO (middle), and LONG ISO (right), post-weaning, plus one additional week of peer rearing, during the baseline period (5 min; upper row; PRE) and during playback (1 min; lower row; DUR) in response to **(A’’)** 50-kHz USV, **(B’’)** 22-kHz USV, and **(C’’)** NOISE, in Experiment 2: Post-weaning Social Isolation - Rescue. The red dashed circles represent 100 % of the total number of arm entries during the baseline period (average over all nine experimental groups). The sizes of the circles depicting proximal (black), neutral (grey), and distal (white) arm entries represent total number of arm entries as percentage of 100 %. *p<0.050 compared to SHORT ISO; ^#^p<0.050 compared to baseline locomotor activity.

**
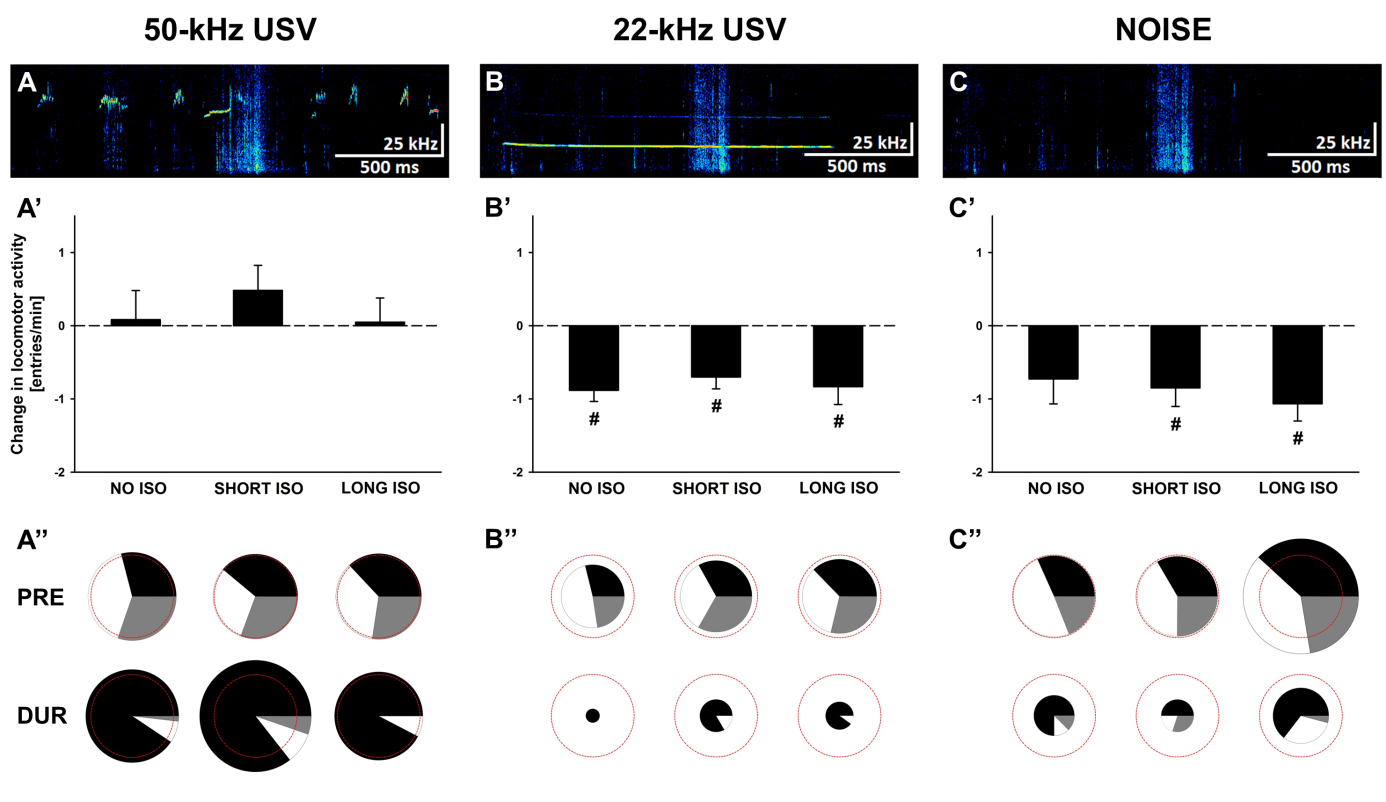
**

**Supplementary Figure 3: Post-adolescent social isolation does not induce behavioral inhibition in response to pro-social 50-kHz USV but in response to alarm 22-kHz USV and NOISE.** **(A)**, **(B)**, and **(C)** Exemplary spectrograms of acoustic stimuli used for playback, namely **(A)** 50-kHz USV, **(B)** 22-kHz USV, and **(C)** background noise (NOISE). **(A’)**, **(B’)**, and **(C’)** Bar graphs depicting changes in locomotor activity as assessed by means of total arm entries per min [entries/min] displayed by rats exposed to four weeks of NO ISO (left), SHORT ISO (middle), and LONG ISO (right), post-adolescent, in response to **(A’)** 50-kHz USV, **(B’)** 22-kHz USV, and **(C’)** NOISE, in Experiment 3: Post-adolescent Social Isolation - Effects. **(A’’)**, **(B’’)**, and **(C’’)** Pie charts depicting changes in stimulus-directed locomotor activity as assessed by means of numbers of proximal (black), neutral (grey), and distal (white) arm entries displayed as percentages by rats exposed to four weeks of NO ISO (left), SHORT ISO (middle), and LONG ISO (right), post-adolescent, during the baseline period (5 min; upper row; PRE) and during playback (1 min; lower row; DUR) in response to **(A’’)** 50-kHz USV, **(B’’)** 22-kHz USV, and **(C’’)** NOISE, in Experiment 3: Post-adolescent Social Isolation - Effects. The red dashed circles represent 100 % of the total number of arm entries during the baseline period (average over all nine experimental groups). The sizes of the circles depicting proximal (black), neutral (grey), and distal (white) arm entries represent total number of arm entries as percentage of 100 %. ^#^p<0.050 compared to baseline locomotor activity.
